# Randomized Pilot Study on the Effects of Sarcocornia as a Salt Substitute in Arterial Blood Pressure and Vascular Function in Healthy Young Adults

**DOI:** 10.3390/foods11182888

**Published:** 2022-09-17

**Authors:** Telmo Pereira, Andreia Torres Caldeira, Armando Caseiro, Nádia Osório, Aida Moreira da Silva, Maria João Barroca

**Affiliations:** 1 Coimbra Health School, Polytechnic of Coimbra, Rua 5 de Outubro—SM Bispo, Apartado 7006, 3046-854 Coimbra, Portugal; 2 Laboratory for Applied Health Research (LabinSaúde), Rua 5 de Outubro—SM Bispo, Apartado 7006, 3046-854 Coimbra, Portugal; 3 Molecular Physical-Chemistry R&D Unit, Department of Chemistry, University of Coimbra, 3004-535 Coimbra, Portugal; 4 Coimbra Agriculture School, Polytechnic of Coimbra, Bencanta, 3045-601 Coimbra, Portugal

**Keywords:** hypertension, arterial stiffness, salt, Sarcocornia, halophyte plants

## Abstract

Previous studies have shown that excessive salt intake is strongly associated with high blood pressure (HT), vascular dysfunction, and the overall risk of cardiovascular diseases. The aim of this study was to evaluate Sarcocornia effectiveness as a salt substitute, addressing its effect on cardiovascular function in healthy young individuals. Thirty healthy participants, aged 18 to 26 years, were randomized into two groups: the control group (CG) and the intervention group (IG). The IG used Sarcocornia powder as a salt substitute for cooking, and the CG used regular salt, during a period of 1 month. A baseline evaluation was performed before the participants started the intervention phase, and was repeated after a 30-day intervention period. Each evaluation included blood pressure (BP) measurement, carotid-femoral pulse wave velocity (PWV), and carotid pulse wave analysis (PWA), and blood samples were also collected for analysis. Sodium excretion was measured at baseline and after intervention through spot urine collection and analysis, a method suitable for this population but with known limitations. Baseline parameters were similar between groups and were within the normal range. Sodium excretion remained unchanged in the two evaluations in the CG, but significantly decreased after intervention in the IG. The reduction in sodium excretion in the IG was followed by a significant reduction in brachial and aortic systolic (SBP) and diastolic blood pressure (DBP), and also in PWV. No significant changes were observed in the CG in terms of cardiovascular parameters. This preliminary study conveys positive results in favor of Sarcocornia as a dietary substitute for regular salt, providing added evidence of the negative cardiovascular effects of high salt intake in young and healthy adults.

## 1. Introduction

Cardiovascular diseases (CVD) constitute the main causes of death and disability, and arterial hypertension (HT) is a major contributor to the overall cardiovascular risk worldwide [1]. Following the current demographic trends towards an ageing society, isolated systolic hypertension (ISH) will be particularly prevalent, reflecting the arterial stiffening that translates the arteriosclerotic phenomena that accompany aging [2,3,4]. Obviously, in less developed societies where saline intake is reduced, the prevalence of HT, even with increasing age, is considerably lower when compared to western countries where salt intake is comparatively larger [4,5].

Sodium plays a fundamental physiological role in terms of body fluids regulation, acid-base balance, nerve impulse conduction, nutrients absorption, kidney function, cardiac output, and muscle contraction [2,5]. However, the physiological sodium requirement of our organism is only 1.2 g/day [2]. According to the World Health Organization (WHO), the recommended amount of salt per day should not exceed 5 g, though current salt consumption is estimated to be around 9–12 g/day in most countries worldwide [6,7].

Excessive salt intake is known for increasing blood pressure (BP), and has an important role in arterial stiffness and vascular aging [4,8,9]. In fact, high salt intake was shown to promote BP increase and stiffening of the elastic arteries, leading to early vascular aging (EVA), which is in turn associated with increased cardiovascular morbidity and mortality due to further systemic hemodynamic changes that include increased pulse pressure (PP), significant wave reflections with higher afterload for the left ventricle, and unbalanced ventricle-arterial coupling with compromised overall cardiovascular efficiency, amongst others [10]. Salt consumption above the recommended limits is responsible for about 9.5% of deaths from cardiovascular causes worldwide and 17.8% of premature deaths from cardiovascular causes [11]. Hence, public health policies aimed at reducing salt intake at the populational level are of the utmost priority [12].

Several studies have demonstrated how the adoption of healthier lifestyles and diets, including the “DASH” (dietary approaches to stop hypertension) and the Mediterranean diet, favor the prevention of HT, contributing to the maintenance of BP values within normal limits [13]. The effectiveness of these dietary approaches is highly dependent on low salt consumption, encompassing the reduction in processed foods congenial to an increased ingestion of essential nutrients and the reinforcement of fruits and vegetables in the diet [14]. Notwithstanding, previous studies have suggested the need to explore alternative strategies to promote a reduction in salt intake [12]. The identification of healthy alternatives to salt has been a fundamental strategic axis, aiming for the identification of compounds that can convey the salty flavor to foods without causing the harmful effects that are recognized in conventional salt [15]. Numerous researchers have reported Salicornia and its similar species, such as Sarcocornia, as a potential alternative. Historically used as a folk medication, Salicornia and Sarcocornia are plants that were recently acknowledged for their nutritional properties and potential for use in the development of functional foods [2,16,17,18]. They appear to be a good alternative to common salt since they have a salty taste and are simultaneously rich in minerals such as magnesium, calcium, iron, potassium, and sodium, and also in polyphenols [17]. The presence of potassium is of particular importance as it improves the excretion of sodium from the body and, therefore, might contribute to reduce the harmful effects of sodium excess [19]. Furthermore, this coastal halophyte is also rich in polyphenols, which are well known protective agents for the cardiovascular and gastrointestinal system, as well as for the control of diabetes and cholesterol [20].

The purpose of this work was to evaluate the cardiovascular benefits of a species of Sarcocornia, as a salt substitute in young and clinically healthy adults, addressing its effects on peripheral and central BP and on aortic pulse wave velocity (PWV) as a surrogate for arterial stiffness [21].

## 2. Materials and Methods

### 2.1. Study Design

**Figure 1:**
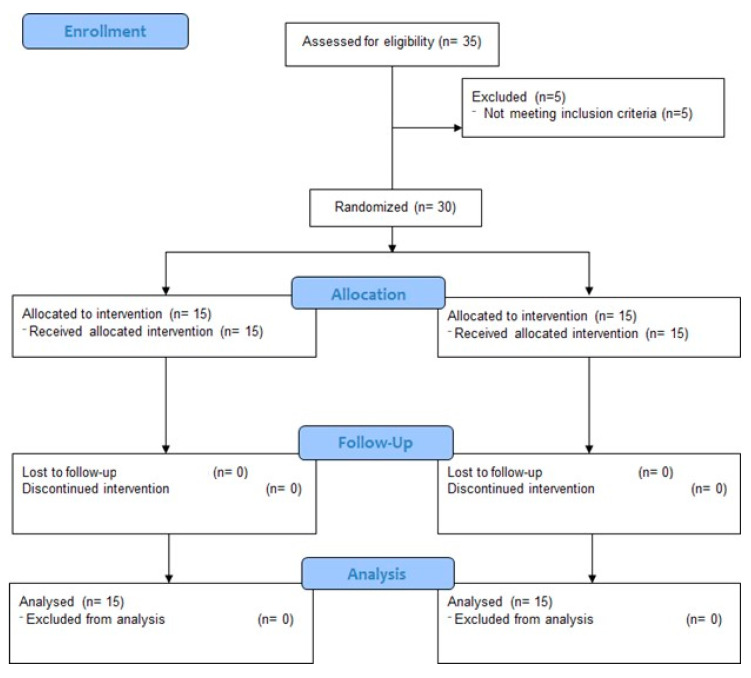
Consort flow diagram outlining the design and conduct of the clinical study.

A randomized, controlled, and double-blind study was carried out to address the effects of Sarcocornia-based salt substitution on the cardiovascular system in young and healthy adults. A total of 30 participants were randomized into two groups (Figure 1), with an allocation ratio of 1:1, through a block randomization procedure, thus ensuring equal sample size groups *(n* = 15 participants per group): control group (CG—15 participants), which maintained the salt intake in their diet; intervention group (IG—15 participants), which used Sarcocornia-based salt in their diet. Each participant received 1000 g of Sarcocornia-salt or regular salt, according to the randomization, and was instructed to use the provided compound in every meal, ensuring an adequate management throughout the 30 days of intervention. The recommendation was to freely use the compounds according to randomization (either regular salt or Sarcocornia-based salt) in food making and overall seasoning in order to guarantee the typical salty flavor of each participant’s diet. Only the lead researchers were aware of the allocation of the participants, which was otherwise concealed for the participants and the researchers performing diagnostic procedures, thus ensuring the double-blind design.

In order to observe the effects of each of the compounds over the 30 days, a baseline assessment was carried out prior to the intervention period and a reassessment of the same parameters was made after the intervention period.

This study was developed at the Coimbra Health School from March 2019 to June 2020. The research project was approved by the Ethics Commission of the Polytechnic Institute of Coimbra (approval 07/2019), and fully complied with the ethical principles of the Declaration of Helsinki. All participants signed their informed consent to participate in the study. The anonymity and confidentiality of the data were ensured. The researchers have no conflicts of interest to declare, and the study was conducted for strictly academic purposes.

### 2.2. Study Population

Sample size was estimated through G*Power sample calculation, setting statistical power at 80%, alpha at 0.05, and a medium effect size. A total of 35 young adults were assessed for elegibility, and 30 were selected to participate in the study, with 5 participants being excluded due to ongoing medication for acute health conditions. The sample included people of both genders (76.7% female), aged between 18 and 24 years (mean age 20.4 ± 1.2 years). The participants were randomly selected within the academic community of a Portuguese university, and were submitted to a pre-study screening to check their compliance with the inclusion criteria and their availability to attend the evaluation moments, as well as their commitment to an adequate use of the product. The inclusion criteria included clinical healthy status, no ongoing medication, both genders, and age between 18 and 35 years old (young adults). Any individuals with a personal history of cardiovascular disease, acute diseases, and known allergies, or under any ongoing medication were excluded.

### 2.3. Halophyte Plant

**Figure 2:**
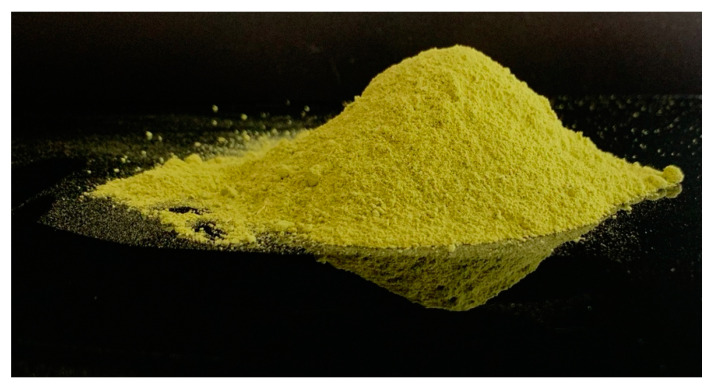
Dried powder of *Sarcocornia perennis*.

*Sarcocornia perennis* (Figure 2) was provided by the Salina Greens company (Alcochete, Setúbal, Portugal). The nutritional and mineral composition of dried *Sarcocornia perennis* (powder) can be found elsewhere [17,22] and are summarized in Table 1.

### 2.4. Endpoints

#### 2.4.1. Aortic Pulse Wave Velocity (PWV)

Aortic PWV is a well-established marker of arterial stiffness (PWV) and predictor of cardiovascular risk [21,23,24,25]. This biomarker shows high values in situations such as HT, aging, physical inactivity, and diabetes [21,23,26]. The measurement of PWV was performed with the Complior^®^ Analyze device (Alam Medical, Saint-Quentin-Fallavier, France) according to current international guidelines [25]. In summary, PWV was assessed non-invasively in the carotid-femoral segment (cfPWV), on the right side of the body, with the participant in a supine position and with the neck slightly hyperextended to the left, after a 10-min rest period. Two probes were positioned over the right carotid and right femoral arteries and pulse waves were simultaneously recorded, when meeting the quality criteria, for a period of 15 s. The time interval between the beginning of the carotid wave and the beginning of the femoral wave was automatically measured by the device, providing the pulse transit time (PTT) for the calculation of the cfPWV. The distance between the two acquisition points was measured with a tape, in a straight line, directly on the body surface. As previously recommended, this distance was then corrected as follows: corrected distance = obtained distance x 0.80 [25]. PWV was calculated as the ratio between the corrected distance and the PTT (in m/s). All determinations were made by a single competent and experienced operator, thus minimizing interoperator variability and improving the reproducibility index.

#### 2.4.2. Carotid Pulse Wave Analysis (PWA)

Carotid PWA was performed with the Complior^®^ Analyze device (Alam Medical, Saint-Quentin-Fallavier, France) simultaneously to the cfPWV acquisition. Before performing PWA and cfPWV, brachial blood pressure (bBP) was measured with a Riester ri-champion^®^ N sphygmomanometer (Riester, Germany; validation reference [27]), with a cuff adjusted to the arm diameter. Systolic (bSBP) and diastolic (bDBP) bBP were obtained and inputted into the Complior^®^ Analyze software. The carotid pressure waveforms, acquired during a 15 s acquisition window, were calibrated with bDBP and brachial mean arterial pressure (bMAP), and their morphology and duration components were automatically estimated, mainly: central systolic blood pressure (cSBP), central PP (cPP), augmentation index (AIx), augmentation index corrected for heart rate (AIx@75), augmentation pressure (AP), left ventricular ejection time (LVET), diastolic filling time (DT), final systolic pressure (PES), sub-endocardial viability ratio (SEVR), and LV contractility index (dP/dt Max).

#### 2.4.3. Assessment of Saline Excretion

The quantification of saline excretion was achieved by performing spot urine laboratory tests at the two assessment times (pre- and post-intervention). This assessment was performed by a competent professional and under appropriate technical conditions. To estimate daily salt intake, a previously validated method was used [28,29], which is based on the estimation of sodium levels and creatinine levels collected in a spot urine sample [28,29]. For this purpose, a urine sample was collected in an appropriate container, preferably the first one in the morning, since it has a higher concentration. From this sample, sodium and creatinine concentrations were determined for the subsequent calculation of 24 h saline excretion. To perform this calculation, the following formula was used:

24 h urinary salt excretion (g/day) = 0.0585 × 21.98 × [UNa[mEq/L]/UCr[mg/L] × (−2.04 × age + 14.98 × weight[kg] + 16.14 × height[cm] − 2244.45)]^0.392^, according to [28,29]. Considering this is a pilot study in young and healthy adults, this method was considered the most suitable, notwithstanding the known limitations.

Procedure:

Figure 1 outlines the design, recruitment and conduct of the study.

Participants volunteering to participate in the study and meeting the inclusion criteria were scheduled for a baseline evaluation during the morning in fasting conditions at the Coimbra Health School laboratories. Informed consent was obtained and questionnaires applied, including a sociodemographic and clinical questionnaire and the Portuguese versions of the Profile of Mood States (POMS) [30] and PREDIMED [31] questionnaires. The POMS questionnaire is a validated psychological test addressing the mood of the participant, containing 65 words or statements (descriptors) that describe feelings people have. The descriptors are punctuated in a Likert scale and clustered in 6 dimensions (Tension, Depression, Anger, Vigor, Fatigue, and Confusion), which are summed to produce an overall mood profile score. The PREDIMED is a 14-item questionnaire that assesses adherence to a Mediterranean diet. The items are punctuated in a Likert scale and a final score of 10 or above provides evidence of a good adhesion to Mediterranean diet. For the assessment of saline excretion through urine, participants were asked to bring their appropriate container with a urine sample, preferably the first one in the morning, since it has a higher concentration, facilitating its analysis. In the Biomedical Science laboratory, a blood sample was taken. Hemodynamic assessments, namely BP and PWV assessment, took place in the Physiology laboratory. At the end of the baseline assessment, and according to the randomization, each participant received a bag containing either regular salt or Sarcocornia powder, and was instructed to use that compound to season their meals over 30 days while maintaining their usual daily routines. The participants were blinded regarding the compound they received, as well as the researchers conducting the physiological assessments. After one month, the procedures were repeated by the same operators and according to the same methodologies.

#### 2.4.4. Statistical Analysis

Data were gathered in a Microsoft Office Excel 2007 program spreadsheet and subsequently exported to the SPSS version 24 program (IBM, Armonk, NY, USA) for statistical analysis. Categorical variables were presented as frequencies and percentages. The normality of the data was confirmed using the Shapiro–Wilks test, and the Levene test was applied to verify the homogeneity of the variances. χ2 or Fisher’s exact tests were used for comparisons involving categorical variables. A mixed-design ANOVA was used to assess the within-subject evolution of the continuous variables under study and to implement between-group comparisons. A further ANCOVA was also implemented in the comparison of the post-intervention of each parameter adjusting to its baseline (included as covariate in the model). Sphericity was assessed using the Mauchly test, and the Greenhouse–Geisser correction was applied when needed. The Bonferroni adjustment was adopted. A *p*-value < 0.05 was considered significant for a 95% confidence interval.

## 3. Results

### 3.1. Baseline Sample Characterization

The sample included a total of 30 healthy young adults of both sexes (76.7% female) aged between 18 and 24 years (mean age 20.4 ± 1.2 years) and with an average BMI of 22.1 ± 2.4 kg/m^2^. The waist and hip circumferences were evaluated, obtaining an average of 76.2 ± 8.1 cm and 94.6 ± 7.9 cm, respectively. Through the application of the PREDIMED questionnaire and considering an individual score of ten or more as good adherence to the Mediterranean-type diet, we found that only one of the participants (3.3% of the sample) followed this type of diet [31], with an average for all individuals of 7.0 ± 1.5. The psychological status of the young people was also quantified, with an average score of 120.0 ± 18.3 on the POMS questionnaire. The levels of happiness and satisfaction presented mean values of 7.1 ± 1.4 and 6.7 ± 1.8, respectively, on a scale from zero to ten. Among the 30 participants, 3 presented cardiovascular risk factors (CVRF), a sedentary lifestyle was the most frequent, and 13 had a cardiovascular history in the family. A single smoker individual was identified, 22 regular coffee consumers, and 28 occasional alcohol consumers. There were no significant differences between the control group and the intervention group according to the baseline assessments performed (Table 2).

### 3.2. Vascular Effects of the Intervention

The analysis of Table 3 allows us to verify that, at baseline level, there were no significant differences between the CG and the IG regarding the values of BP and HR. After 30 days of intervention, these parameters were again determined, and we observed that the individuals who integrated the IG obtained lower values of brachial BP. This reduction occurred in bSBP, where the greatest difference was observed (−7.0 ± 0.9 mmHg), in bDBP, bMBP, and in bPP, although in this last parameter the difference was not significant. In the CG, which maintained salt intake in their diet, there were no significant variations. The individuals who were part of the IG showed a significant increase in HR from the first to the second moment.

Table 4 shows the results of central arterial hemodynamics that were obtained by comparing the two groups before and after the intervention period. We verified that the cfPWV and cSBP values experienced a significant reduction from moment one to moment two in the IG. The difference observed in cfPWV was −0.2 ± 0.1 m/s (*p* = 0.042), and in cSBP it was −5.6 ± 1.0 mmHg (*p* < 0.001). Although the significance criterion (*p* < 0.05) was not met in either group, the AIx decreased in both groups with a more marked reduction in the IG. The cPP, AP, SP Amp, and PPratio values did not differ between the two moments in either group. Regarding the sub-endocardial viability index (SEVR), there was a significant growth from moment one to moment two in the IG: at moment one, it presented the value of 132.6 ± 30.1, while at moment two, the value of 151.3 was determined to be ± 22.3 (difference = 18.7 ± 4.7 and *p* = 0.001). Regarding the left ventricular contractility index (max dP/dT), an increase in the mean value in the IG was observed, with the significance criterion close to the defined statistical significance criterion (*p* = 0.057). The main effects were further explored in an ANCOVA analysis addressing post-intervention differences adjusted to the baseline data (included as covariates), in which no effect modification was depicted in line with the results portrayed in Table 3 and Table 4.

### 3.3. Effects of the Intervention on Salt Excretion

**Figure 3:**
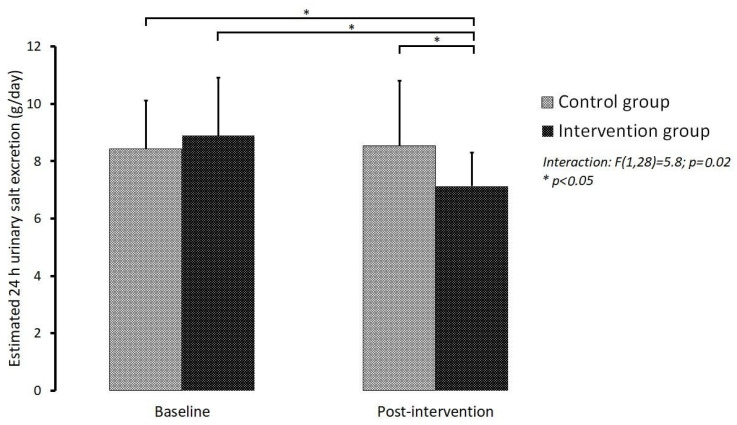
Comparison of daily saline excretion between groups at the two assessment times (moment 1: pre-intervention; moment 2: post-intervention).

The statistical information collected allowed the elaboration of the graph represented in Figure 3, where the results obtained in relation to the saline excretion in the two moments of evaluation are compiled, comparing their evolution in both groups. From their analysis, we verified that the IG and the CG showed a different evolution of saline excretion between the two moments. The CG maintained similar mean values at both moments: 8.4 ± 1.8 g/day at moment one and 8.5 ± 2.4 g/day at moment two. In the IG, there was a significant reduction in values: 8.9 ± 2.1 g/day at moment one and 7.2 ± 1.2 g/day in moment two, with a mean difference of −1.7 ± 1.6 g/day (*p* = 0.002). The existence of a significant interaction Group * Moment (F,interaction (1,28) = 5.8; *p* = 0.02) indicates that the behavior (variation) of saline excretion from one moment to the next is different depending on the group: stabilization in the control group and reduction in the intervention group. The average daily saline excretion in the total population was 8.7 ± 1.9 g at baseline and 7.8 ± 2.0 g/day in the post-intervention assessment. It should also be noted that there are no significant differences in the comparison of baseline values between groups.

## 4. Discussion and Conclusions

This pilot randomized trial was aimed at ascertaining the cardiovascular benefits of a species of Sarcocornia as a salt substitute in young and clinically healthy adults, with a particular emphasis on peripheral and central BP and on aortic pulse wave velocity (PWV) as a surrogate for arterial stiffness. Our results demonstrated that Sarcocornia is an effective salt substitute, and is associated with beneficial cardiovascular effects, such as BP lowering and PWV improvement in young and healthy adults.

Although salt (sodium chloride) plays an important role as a fluid regulator in our body, there are several studies that point to its harmful effects on the cardiovascular level when consumed in excess. A series of research works have been carried out in search of a substitute for common salt in order to try to reduce its consumption [2,7,9,15,20,22,32,33,34,35,36]. Halophyte plants, such as Salicornia and Sarcocornia, prove to be a promising substitute, given that they are plants with antihypertensive characteristics [15,35]. Their high content of minerals such as potassium and magnesium favors saline excretion, reducing the negative effects of sodium on the cardiovascular system and the human body in general [18,22,35]. These plants are part of the native Portuguese flora and grow in abundance in coastal salt pans, which gives them a salty taste favorable to the adaptation of the palate [7,16,17,18,33].

In this study, as previously shown in other investigations, the beneficial properties of Salicornia and Sarcocornia for the cardiovascular system were confirmed. Although these investigations were carried out, mostly, in animal species, the results obtained in this study with the human species are similar. A significant reduction in BP was observed with the ingestion of Sarcocornia instead of salt. These results are in line with the research carried out on normotensive Sprague Dawley (SD) rats by Panth et al. in 2016 [2]. Only one difference is observable in terms of DBP, since in the present study there was a significant reduction with the use of Sarcocornia, while in the research mentioned above, the observed reduction did not reach the criteria of significance. Another investigation carried out in SD rats, this time by Zhang et al. in 2015, argued that the intake of Salicornia does not reduce the levels of BP, it only prevents the development of HT, since it does not cause an increase in BP when compared with salt intake [15]. In view of these results, we continue to consider Sarcocornia as an interesting substitute for the sodium chloride commonly used.

Regarding the HR, no studies were found to investigate the influence of Sarcocornia. According to the results obtained in the present study, HR increased significantly in individuals submitted to Sarcocornia intake. This behavior is most likely due to the reduction in BP levels and the triggering of the baroreceptor reflex, which is probably a compensatory response that increases the sympathetic response, promoting an increase in HR, cardiac output, and myocardial contractility [36]. On the other hand, the improvement in vascular function, with a greater relaxation of the arterial wall that explains the improvement observed in PWV in IG, can contribute to an optimization in the coupling between the left ventricle and the aorta, resulting in greater mechanical and hemodynamic efficiency, which may explain the improvement trend observed in the dP/dT and the significant improvement in the sub-endocardial viability index identified in the IG. The improvement in the PWV observed in the IG, which is a biomarker recognized for its relationship with cardiovascular risk [21], suggests that Sarcocornia may play an important role in vasoprotection, promoting its distensibility and elasticity. On the other hand, the reported positive modulation verified in the sub-endocardial viability index indicates an optimization of coronary perfusion and arterial load [37]. All these data support the idea that Sarcocornia substantially favors the entire cardiovascular system.

In this study, the daily saline excretion between groups was also compared at the two assessment times, and through its results we can observe a significant reduction in the levels of sodium in the urine of individuals belonging to the intervention group. Several studies have indicated that Sarcocornia is a halophyte rich in minerals such as magnesium and potassium, which improve the body’s sodium excretion, thus supporting the data obtained in the present investigation [18,22].

Despite the very promising results, it is important to note that this is a pilot trial and several limitations must be considered in this study. Among the main limitations are the small sample size and the short intervention duration. The small sample size increases the risk for information and selection bias, thus recommending caution in the generalization of the results. Also remarkable is the impossibility of controlling the proper use of salt and Sarcocornia and the daily diet by the participants. However, the estimation of sodium partially overcomes this limitation. Future studies should include the use of robust nutritional questionnaires, such as the food frequency questionnaire and the 24-h recall, to add further nutritional control in the comparisons between groups. Even though the spot urine method has been used in previous studies and has been validated [27,28], it has limitations that recommend caution in the interpretation of the results. In future research, 24-h urine collection should be employed for better characterization of salt excretion. Participants reported that Sarcocornia had a reduced taste and gave an intense green color to some foods. The changes observed in PWV in such a short duration intervention period should reflect functional adaptations rather than structural changes, which would be expected to occur for longer intervention periods. It should also be borne in mind that all of these assessments were carried out on healthy young adults, so the effect cannot be extrapolated to other clinical contexts, particularly HT. However these results are encouraging for the replication of this study in different populations, involving adults with risk factors such as AHT, diabetes, and dyslipidemia, as well as in children.

In summary, based on the results of the present study, the consumption of Sarcocornia is beneficial to the cardiovascular system at several levels: attenuation of blood pressure values, decreased arterial stiffness, improved left ventricular function and coronary perfusion. This halophyte and similar species have already been tested and approved in the production of several processed foods, namely ham, snacks, sausages, and bread [7,22,34,37], since these are the foods that contain the highest sodium content. Sarcocornia can therefore be considered a good substitute for common table salt to try to reduce the prevalence of HT and deaths from cardiovascular causes, although further studies should be carried out to investigate the remaining species of this plant and also to evaluate the amount ideal to be ingested, since its excessive consumption may cause possible harmful effects or even influence some types of medication [38]. Another future challenge is the development of Sarcocornia formulations that facilitate its use in the substitution of common table salt and that eliminate some of the constraints associated with its use in the current formulation, namely regarding the texture and coloring.

## Figures and Tables

**Table 1:** Proximate biochemical composition (g/100 g) and mineral composition (mg/100 g) of dried *S. perennis* (powder) [17,22].

**Biochemical Compounds**	**g/100 g**
Crude protein	16.7 ± 0.1
Total fat	2.3 ± 0.8
Crude fiber	9.5 ± 0.1
Total ash	33.9 ± 0.1
Carbohydrates	39.5
**Minerals**	**mg/100 g**
Na	7119.35 ± 195.12
K	1830.54 ± 49.15
Ca	490.87 ± 10.02
Mg	786.39 ± 20.94
P	230.56 ± 2.32
Fe	8.64 ± 0.18
Cu	0.68 ± 0.01
Zn	2.45 ± 0.03
Mn	3.49 ± 0.04

**Table 2:** Characterization of the study population.

	Total (*n* = 30)	CG (*n* = 15)	IG (*n* = 15)	*p*-Value
Age, years	20.4 ± 1.2	20.6 ± 1.5	20.2 ± 0.9	0.379
Female gender, %(*n*)	76.7 (23)	80.0 (12)	73.3 (11)	0.666
BMI, Kgm^−2^	22.1 ± 2.4	22.8 ± 1.9	21.3 ± 2.7	0.085
Waist, cm	76.2 ± 8.1	74.6 ± 8.0	77.8 ± 8.4	0.296
Hip, cm	94.6 ± 7.9	94.4 ± 8.4	94.8 ± 7.9	0.894
DM Adherence	7.0 ± 1.5	7.3 ± 1.4	6.6 ± 1.7	0.205
Smokers, %(*n*)	3.3 (1)	0.0 (0)	3.3 (1)	0.309
Happiness	7.1 ± 1.4	6.8 ± 1.6	7.3 ± 1.3	0.318
Satisfaction	6.7 ± 1.8	6.2 ± 2.0	7.1 ± 1.4	0.156
POMS	120.0 ± 18.4	121.8 ± 20.9	118.3 ± 16.7	0.613

BMI, body mass index; DM adherence, adherence to the Mediterranean diet; CG, control group; IG, intervention group.

**Table 3:** Mean values of blood pressure and heart rate at moment 1 (baseline) and moment 2 (post-intervention) in both groups, showing the evolution between the 2 moments.

		CG (*n* = 15)	IG (*n* = 15)	*p*-Value
bSBP (mmHg)	Moment 1	112.1 ± 7.7	111.3 ± 8.5	0.789
	Moment 2	114.4 ± 7.2	104.3 ± 6.1	<0.001
	Difference	2.3 ± 1.6	−7.0 ± 0.9	
	*p*-value	0.169	<0.001	
bDBP (mmHg)	Moment 1	63.7 ± 5.2	65.9 ± 4.8	0.235
	Moment 2	64.6 ± 4.9	60.0 ± 2.7	0.004
	Difference	0.1 ± 1.2	−5.9 ± 1.0	
	*p*-value	0.491	<0.001	
bPP (mmHg)	Moment 1	48.5 ± 6.8	45.5 ± 11.0	0.378
	Moment 2	49.9 ± 7.0	44.3 ± 7.1	0.040
	Difference	1.4 ± 1.1	−1.1 ± 1.4	
	*p*-value	0.229	0.426	
bMBP (mmHg)	Moment 1	79.8 ± 5.2	80.9 ± 3.3	0.506
	Moment 2	81.1 ± 4.7	74.7 ± 2.4	<0.001
	Difference	1.3 ± 1.3	−6.1 ± 0.7	
	*p*-value	0.313	<0.001	
HR (bpm)	Moment 1	68.7 ± 12.0	66.0 ± 8.8	0.484
	Moment 2	71.1 ± 17.1	70.5 ± 10.9	0.920
	Difference	2.3 ± 2.4	4.5 ± 1.5	
	*p*-value	0.355	0.011	

bSBP, brachial systolic blood pressure; bDBP, brachial diastolic blood pressure; bPP, brachial pulse pressure; bMBP, brachial mean blood pressure; HR, heart rate.

**Table 4:** Difference between the control group and the intervention group in the 2 assessment moments in the central hemodynamic variables.

		CG (*n* = 15)	IG (*n* = 15)	*p*-Value
cfPWV (m/s)	Moment 1	5.9 ± 0.9	6.0 ± 0.6	0.282
	Moment 2	6.1 ± 1.0	5.7 ± 0.6	0.238
	Difference	0.2 ± 0.2	−0.2 ± 0.1	
	*p*-value	0.186	0.042	
cSBP (mmHg)	Moment 1	106.7 ± 9.2	104.8 ± 9.1	0.882
	Moment 2	108.0 ± 7.3	99.2 ± 8.4	0.005
	Difference	1.3 ± 2.6	−5.6 ± 1.0	
	*p*-value	0.631	<0.001	
cPP (mmHg)	Moment 1	43.1 ± 9.6	38.9 ± 10.9	0.600
	Moment 2	43.5 ± 7.2	39.2 ± 9.6	0.180
	Difference	0.4 ± 2.3	0.3 ± 1.2	
	*p*-value	0.864	0.827	
AIx@75 (%)	Moment 1	−22.0 ± 17.8	−30.8 ± 23.7	0.055
	Moment 2	−23.6 ± 17.7	−33.7 ± 21.3	0.168
	Difference	−1.5 ± 3.6	−2.9 ± 5.1	
	*p*-value	0.674	0.581	
AP (mmHg)	Moment 1	9.5 ± 9.0	11.3 ± 10.4	0.109
	Moment 2	10.1 ± 9.7	13.4 ± 10.7	0.387
	Difference	0.6 ± 1.8	2.0 ± 2.3	
	*p*-value	0.741	0.401	
SPAmp (mmHg)	Moment 1	5.4 ± 8.4	6.5 ± 4.8	0.243
	Moment 2	6.4 ± 4.7	5.1 ± 4.7	0.465
	Difference	1.0 ± 2.3	−1.4 ± 1.2	
	*p*-value	0.675	0.243	
PP ratio (mmHg)	Moment 1	1.2 ± 0.2	1.2 ± 0.1	0.669
	Moment 2	1.6 ± 0.1	1.2 ± 0.1	0.935
	Difference	0.0 ± 0.1	−0.0 ± 0.0	
	*p*-value	0.990	0.272	
SEVR (%)	Moment 1	141.7 ± 28.2	132.6 ± 3.1	0.006
	Moment 2	142.9 ± 39.4	151.3 ± 22.3	0.477
	Difference	1.2 ± 5.7	18.7 ± 4.7	
	*p*-value	0.837	0.001	
Max dP/dT (mmHg/s)	Moment 1	923.3 ± 164.4	842.0 ± 159.2	0.651
	Moment 2	938.7 ± 183.5	873.3 ± 157.9	0.305
	Difference	15.3 ± 54.3	31.3 ± 15.1	
	*p*-value	0.782	0.057	

cSBP, central systolic blood pressure; cPP, central pulse pressure; AIx@75, augmentation index corrected for heart rate; AP, augmentation pressure; SP Amp, systolic pressure amplification; PP ratio, pulse pressure amplification; SEVR, sub-endocardial viability index; Max dP/dT, left ventricular contractility index.

## Data Availability

Data are contained within the article.
